# Evaluating the Potential of Delta Radiomics for Assessing Tyrosine Kinase Inhibitor Treatment Response in Non-Small Cell Lung Cancer Patients

**DOI:** 10.3390/cancers15215125

**Published:** 2023-10-24

**Authors:** Ting-Wei Wang, Heng-Sheng Chao, Hwa-Yen Chiu, Yi-Hui Lin, Hung-Chun Chen, Chia-Feng Lu, Chien-Yi Liao, Yen Lee, Tsu-Hui Shiao, Yuh-Min Chen, Jing-Wen Huang, Yu-Te Wu

**Affiliations:** 1 School of Medicine, National Yang Ming Chiao Tung University, Taipei 112, Taiwan; 2 Institute of Biophotonics, National Yang Ming Chiao Tung University, Taipei 112, Taiwan; 3 Department of Chest Medicine, Taipei Veterans General Hospital, Taipei 112, Taiwan; 4 Department of Radiation Oncology, Taichung Veterans General Hospital, Taichung 407, Taiwan; 5 Department of Biomedical Imaging and Radiological Sciences, National Yang Ming Chiao Tung University, Taipei 112, Taiwan

**Keywords:** time-variable radiomics, computer tomography (CT) scans, progression-free survival (PFS), lung adenocarcinoma, epidermal growth factor receptor-tyrosine kinase inhibitors (EGFR-TKI), delta radiomics signatures, personalized treatment strategies

## Abstract

**Simple Summary:**

In our research, we analyzed the CT scans of 322 advanced lung cancer patients over time to see how long they might remain disease-free after undergoing a specific treatment called EGFR-TKI. By integrating the patterns from these scans with other medical data, such as gene mutations and treatment strategies, we improved our ability to predict the course of the disease. However, when we included data from multiple centers, the consistency of our findings reduced. Simply put, our technique can offer doctors a glimpse into the future progression of lung cancer, and aid in tailoring treatments. This approach could be groundbreaking in lung adenocarcinoma treatment, but it needs further investigation.

**Abstract:**

Our study aimed to harness the power of CT scans, observed over time, in predicting how lung adenocarcinoma patients might respond to a treatment known as EGFR-TKI. Analyzing scans from 322 advanced stage lung cancer patients, we identified distinct image-based patterns. By integrating these patterns with comprehensive clinical information, such as gene mutations and treatment regimens, our predictive capabilities were significantly enhanced. Interestingly, the precision of these predictions, particularly related to radiomics features, diminished when data from various centers were combined, suggesting that the approach requires standardization across facilities. This novel method offers a potential pathway to anticipate disease progression in lung adenocarcinoma patients treated with EGFR-TKI, laying the groundwork for more personalized treatments. To further validate this approach, extensive studies involving a larger cohort are pivotal.

## 1. Introduction

The treatment landscape for non-small cell lung cancer (NSCLC) has experienced significant advancements over the past decade, largely driven by the identification of actionable molecular alterations and the subsequent development of targeted therapies, including tyrosine kinase inhibitors (TKIs) [1,2]. Intriguingly, there have been revelations indicating enhanced prognosis when conventional treatments are complemented with traditional Chinese medicine [3,4]. Despite these advances, accurately assessing treatment responses to TKIs remain critical challenges for the management of patients with NSCLC [5]. Conventional imaging methods such as computed tomography (CT), magnetic resonance imaging (MRI), and positron emission tomography (PET) have limitations in capturing the complexities of tumor biology and its response to targeted therapies [6,7].

To overcome the limitations of traditional imaging methods, a novel approach called “radiomics” has emerged. Radiomics involves the high-throughput extraction of quantitative features from medical images, providing insights into tumor phenotypes and their association with clinical outcomes such as treatment response, prognosis, and disease progression [8,9,10]. Radiomics has shown promise in various cancer types, including NSCLC, by allowing the non-invasive evaluation of tumor heterogeneity, which is often an important determinant of treatment response [11,12]. To ensure reproducibility and comparability across different studies, software platforms, and imaging modalities, the Image Biomarker Standardization Initiative (IBSI) was established. This collaborative effort aims to establish standardized definitions, nomenclature, and reporting guidelines for radiomic features, providing a comprehensive reference manual containing detailed descriptions of radiomic features and their calculations, as well as recommendations for image pre-processing and quality control [13]. Additionally, delta radiomics, which focuses on the temporal changes in radiomic features, has demonstrated potential in improving the sensitivity and specificity of treatment response assessment in cancer patients, as highlighted by several studies [14,15].

EGFR-TKI therapy represents a paradigm shift in the treatment of non-small cell lung cancer (NSCLC). The epidermal growth factor receptor (EGFR) is a transmembrane protein that, when mutated, can promote rapid cell proliferation and tumor progression in NSCLC. Specifically targeting these mutations, EGFR-TKIs (Tyrosine Kinase Inhibitors) have shown significant efficacy in patients, offering improved progression-free survival and overall response rates compared to traditional chemotherapies. The journey started with first-generation TKIs like erlotinib and gefitinib, which demonstrated marked effectiveness against tumors harboring EGFR exon 19 deletions or exon 21 (L858R) substitution mutations [16]. However, resistance, often due to the emergence of the T790M mutation, led to the development of second- and third-generation TKIs. Osimertinib, a third-generation TKI, has shown substantial activity against T790M-positive NSCLC and boasts a better side-effect profile [17]. With the evolving landscape of targeted therapies, EGFR-TKI therapy underscores the importance of molecular profiling in NSCLC to tailor treatments to individual patients.

Recent studies have applied delta radiomics to investigate treatment response in NSCLC patients receiving TKIs or immunotherapy and reported promising results [18,19,20]. However, the field of delta radiomics is still in its infancy, and several challenges need to be addressed to ensure the robustness and reproducibility of the findings, such as standardization of image acquisition, pre-processing, and feature extraction [21,22]. In addition to addressing methodological challenges, the application of advanced machine learning and artificial intelligence techniques can further enhance the predictive power of delta radiomics by identifying complex patterns and interactions between radiomic features and clinical variables [23,24].

Our study aimed to investigate the potential of a time-variable radiomics signatures derived from time-serial CT scans to accurately predict progression-free survival (PFS) and stratify the risk of acquired resistance in lung adenocarcinoma patients undergoing EGFR-TKI treatment. Another key aspect of this study was the comprehensive evaluation of prognostic factors in NSCLC patients receiving EGFR-TKI therapy. We integrated extensive clinical data, including EGFR gene mutation status, TKI usage, and patient clinical staging, along with laboratory data, to enhance the predictive performance of PFS.

## 2. Materials and Methods

### 2.1. Patient Population and Selection Criteria

This study retrospectively included 226 NSCLC patients treated with targeted therapy at Taipei Veterans General Hospital between 2018 and 2019. The patient dataset was collected in accordance with the following inclusion criteria: (1) having more than stage IIIB NSCLC in accordance with the eighth edition of the American Joint Committee on Cancer staging system [25], (2) having pathologically confirmed NSCLC based on molecular examination of surgical or tissue biopsy specimens, (3) receiving first- and second-generation EGFR–TKIs in accordance with the NCCN treatment guidelines [26], (4) having high-quality contrast computed tomography (CT) findings of the chest before dosing and 6 to 16 weeks after dosing, and (5) having complete clinical information. This study was approved by the Institutional Review Board, which waived the requirement for informed consent.

To ensure data consistency and quality, several exclusion criteria were applied in the study. A detailed explanation of the exclusion criteria is provided in the Supplementary Materials. Patients with mutations other than EGFR mutations, ALK fusion, KRAS mutations, or BRAF mutations, as well as those who received third-generation EGFR-TKIs as the first-line therapy, had no visible tumor lesions on images, or had insufficient follow-up information were excluded. In total, 23 ALK-positive patients, one BRAF-positive patient, and nine patients treated with Osimertinib were excluded, as were patients with missing dosing time or clinical data, lost follow-ups, or who had no lesions. Additionally, patients who experienced early death, early progression disease, or follow-up CT scans that were not between 6–16 weeks were excluded. A second validation dataset of 96 NSCLC patients treated with targeted therapy at Taichung Veterans General Hospital between 2018 and 2019 was obtained with the same inclusion and exclusion criteria.

The Institutional Review Board of Taipei Veterans General Hospital and Taichung Veterans General Hospital approved the retrospective study (2021-09-009BCF) and waived the need for informed patient consent. The study was conducted in accordance with the declaration of Helsinki.

### 2.2. CT Data and Image Preprocessing

All those who were eligible underwent baseline chest CT scans within 2 months before and 6 to 16 months after the EGFR-TKI therapy. The study used progression-free survival (PFS) as the primary endpoint, defined as tumor growth, metastasis, adverse reactions necessitating a change in treatment regimen, or patient death. Several preprocessing steps were performed on acquired CT images before the subsequent radiomics analysis. First, the resolution of the CT was adjusted to be the same with a pixel size of 1 × 1 × 1 mm^3^. Secondly, the intensities of the CT were converted into normalized ranges (Z-core transformation) on the basis of the mean and standard deviation of the image set. Finally, low-pass (L)- and high-pass (H)-dimensional wavelet filters were applied to the three axes of the CT to produce eight image sets: LLL, LLH, LHL, LHH, HHL, HLH, HLL, and HHH wavelet-filtered images.

### 2.3. Radiomic Feature Extraction

A team of experienced radiologists and certified pulmonologists evaluated the quality of the CT images and identified regions of interest (ROIs) for analysis. For the purpose of this study, primary tumors were segmented separately from metastatic lesions, and only the primary tumors were included in the ROI analysis. Soft-tissue and lung CT images were used for ROI delineation, with soft-tissue settings used to identify tumors, lung collapse, and fluid components, and lung settings used to identify tumor boundaries.

Radiomic features, including histograms and geometric and texture features (GLCM, GLRLM, and LBP), were extracted from all image sets, including the eight wavelet decomposition images and original CT images. GLCM and GLRLM values were aggregated by averaging the three-dimensional orientation matrix for optimal rotation invariance during feature extraction. LBP features were computed slice by slice, and a histogram analysis of the LBP matrices of all CT and MRI slices was performed. A total of 593 radiological features were generated for each primary tumor ROI [27,28]. All image preprocessing procedures and subsequent radiomics analyses were performed using established platforms and adhered to the IBSI standards [13]. Table S1 lists the formulas used for the radiomics analysis.

To ensure the reliability and reproducibility of the radiomic features, two team members conducted a test–retest analysis by performing segmentations on 30 randomly selected patients. An interclass correlation coefficient (ICC) greater than 0.80 was deemed to indicate excellent reliability and was used to exclude features with low intra-observer agreement. Finally, the remaining features were used to calculate delta radiomic features based on following formula.

(1) $$\mathrm{Delta}\;\mathrm{radiomics}=(\frac{Radiomics\;follow-Radiomics\;pretreat}{Radiomics\;pretreat})$$

(2) $$\mathrm{Delta}\;\mathrm{radiomics}=\frac{1}{Time\;difference}\;\times \;(\frac{Radiomics\;follow-Radiomics\;pretreat}{Radiomics\;pretreat})$$

(3) $$\mathrm{Delta}\;\mathrm{radiomics}=\frac{1}{Time\;difference}\;\times \;(\log\;(Radiomics\;follow)-\log\;(Radiomics\;pretreat))$$

### 2.4. Feature Selection and Predictive Modeling

The TVGH dataset was partitioned using the hold-out method, allocating 70% of the patients to the training set and the remaining 30% to the test set. To investigate the dataset’s applicability in another center, an experiment was conducted by combining the TVGH and TCGH datasets. This combined set was then divided using the same 70–30% ratio. Missing values in laboratory data are imputed using the missing forest method [29]. To identify essential clinical features while minimizing redundancy for progression-free survival (PFS) prediction, a two-stage feature selection strategy was executed on the training dataset. The initial statistical analyses incorporated dummy encoding to transform categorical variables, which were subsequently combined with continuous variables and subjected to univariate Cox proportional hazard regression. A significance level of *p* < 0.1 served as the selection criterion during the first stage. In the second stage, the selected features were input into a multivariate Cox proportional hazard regression model, retaining variables with *p* < 0.1 for further analysis.

For radiomic variables, a three-stage feature selection strategy was applied to the training dataset. The first stage involved comparing pretreatment radiomics, follow-up radiomics, and delta radiomics utilizing Equations (1)–(3). The preliminary statistical tests employed a significance threshold of *p* < 0.05 in the Cox proportional hazard regression model. In the second stage, features with a variance inflation factor greater than five were eliminated. In the third stage, the selected features were input into a multivariate Cox proportional hazard regression model, and the top five features with the largest coefficients were chosen to train a CoxPH model. The results were evaluated by comparing the area under the curve (AUC) at the median PFS (383 days) using 5-fold cross-validation, repeated 10 times.

To ascertain the optimal feature selection and machine learning model, a two-stage feature selection strategy was implemented on the training dataset. The preliminary statistical tests employed a significance threshold of *p* < 0.05 in the Cox proportional hazard regression model. Subsequently, an evaluation comprising four feature selection algorithms (Kbest, Lasso, Ridge, Elastic net) was conducted in tandem with five machine learning models (CoxPH, Survival tree, Random survival forest, Fast SVM, Gradient boosting tree) to identify the optimal performance model containing five radiomic features. The final selected radiomics features were again selected with *p* < 0.1 in multivariate CoxPH model. A correlation matrix using Pearson correlation coefficients was plotted to analyze the associations between clinical and radiomic features.

**Figure 1:**
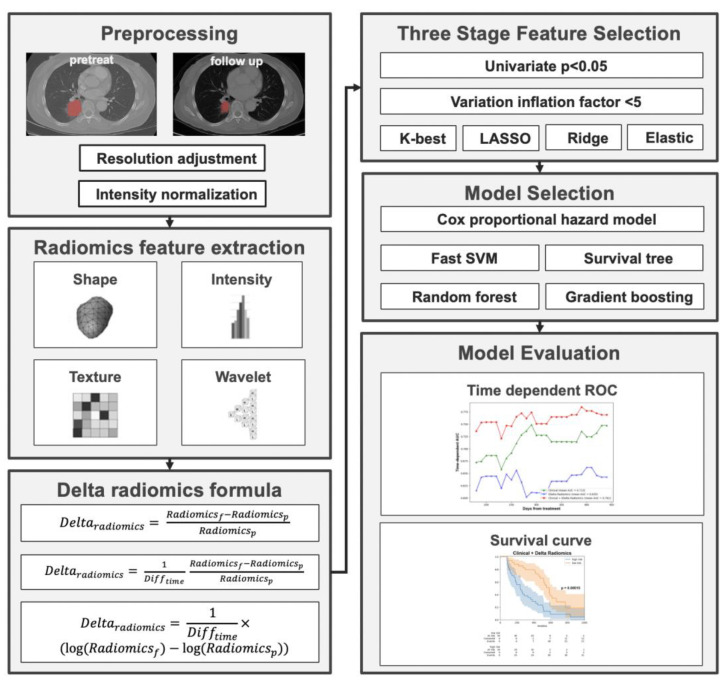
Overall workflow of the study.

The utilization of the Youden index in this context is pivotal for stratifying patients based on their likelihood of disease progression, especially in a scenario where timely intervention can influence outcomes. By leveraging the ROC curve generated from the training set, the Youden index provided a classification threshold for predicting outcomes. Applying this threshold, both the training and validation data were categorized into high-risk and low-risk groups. Kaplan–Meier curves, renowned for their efficacy in survival analysis, were then crafted using the event time data, underscoring the differences between these two risk groups. Specifically, those in the “high-risk” group demonstrated a shorter progression-free survival (PFS), indicating a more rapid disease progression. In contrast, the “low-risk” group showcased a longer PFS, suggesting a prolonged period without disease progression, thereby implying a more favorable response to the given treatment. The overall workflow of the study is shown in Figure 1.

### 2.5. Statistical Analysis

The chi-squared test was used to assess the statistical differences of categorical variables between the training and testing sets. The log-rank test was employed to assess the statistical differences between the high-risk and low-risk groups based on the optimal threshold ascertained from the training set of included patients. The statistical power of the log-rank test was calculated considering an α of 0.05, the estimated hazard ratio, and the sample size. Time-dependent receiver operating characteristic (ROC) curves, the area under the ROC curve (AUC), and the concordance index (C-index) were estimated to evaluate the predictive performance of survival status at various time points, namely 6, 9, 12, and 15 months. A bootstrap resampling technique, in conjunction with the paired *t*-test, was utilized to perform statistical comparisons of the predictive efficacy among the five radiomic aggregation methods. Both the log-rank tests and paired *t*-tests were two-sided, with statistical significance established at a *p*-value of 0.05 or lower. The log-rank test with a significance level of 0.05 was used to determine the differences between survival curves.

## 3. Results

### 3.1. Demographic and Clinical Characteristics of the Patient Cohort

Out of a total of 322 patients, 158 were categorized into the training group, 68 into the test group, and 96 into the external validation group (Table 1). Demographic and clinical characteristics varied across these groups. Females constituted 62.7% of the training group, a notable difference from the 51.5% in the test group and 59.4% in the external group. As for smoking status, 22.2% of the training group were smokers, while the test and external groups reported 23.8% and 21.9% smokers, respectively. In the context of the ECOG PS scores, the training group demonstrated a distribution where 31.0% had a score of 0, 57.6% a score of 1, 7.0% a score of 2, and a minority of 4.4% surpassed a score of 2. In comparison, in the test group, 51.5% scored a 0, 41.2% a 1, 5.9% a 2, and 1.5% had scores exceeding 2. Meanwhile, the external group had 4.2% of patients with a score of 0, a majority of 67.7% with a score of 1, 10.4% with a 2, and a significant 17.6% with scores above 2. Histologically, adenocarcinoma was the predominant form of NSCLC in all groups: 98.1% in the training group, 95.6% in the test group, and 92.7% in the external group. Squamous cell carcinoma was minimal with only 1.3% in the training group, 2.9% in the test group, and absent in the external group.

### 3.2. Comparison of Different Radiomics Methods

Table 2 presents the performance evaluation of various radiomic approaches, each utilizing five radiomic features in conjunction with the CoxPH model. The findings indicate that the delta radiomics method outperformed both the pretreatment and follow-up radiomics methods in terms of C-index and AUC. Specifically, among the delta radiomics approaches, the formula derived from the percentage change of radiomic features demonstrated superior results, yielding a validation C-index of 0.58 and a time-dependent AUC of 0.60.

### 3.3. Final Selected Features Included in the Model

**Figure 2:**
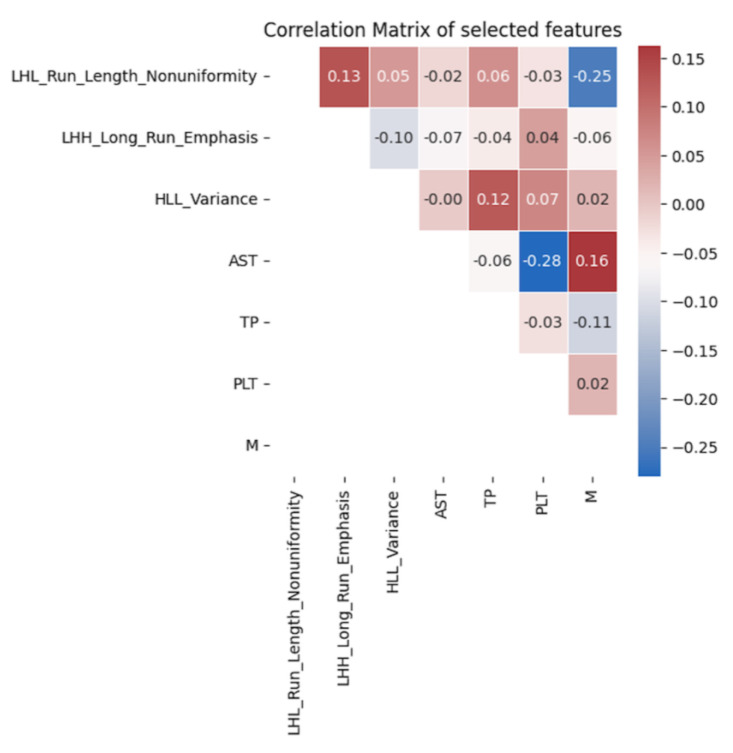
Correlation analysis of the final selected features.

For clinical features, the selected variables included N staging, M staging, platelet count, aspartate aminotransferase (AST), and total protein. For delta radiomics features, LHL_Run_Length_Nonuniformity, LHH_Long_Run_Emphasis, and HLL_Variance were chosen. These variables exhibited significant correlations with progression-free survival in both univariate and multivariate analyses, as shown in Table 3. Additionally, Figure 2 demonstrates that the selected delta radiomics features did not exhibit high correlations with the clinical features. In the context of the CoxPH model, the low correlation between delta radiomics and clinical features implies that the model’s predictive performance is not unduly influenced by multicollinearity, thus ensuring the independence and reliability of the selected variables in predicting progression-free survival.

### 3.4. Performance of Predictive Models for the Prediction of Progression Free Survival

**Figure 3:**
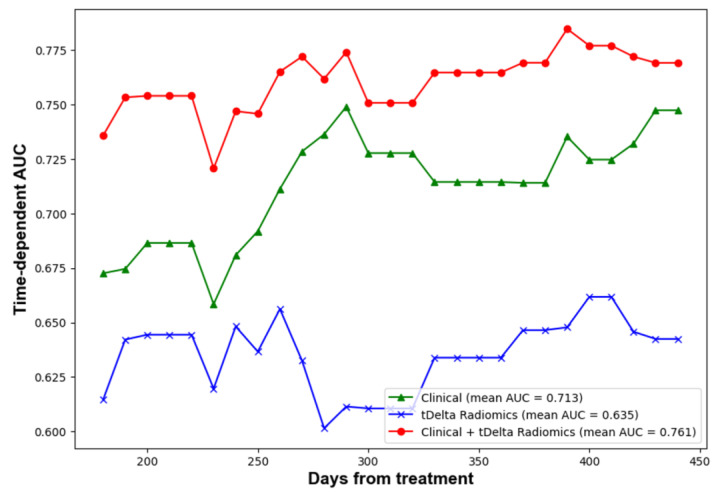
Time dependent AUC of performance of clinical, radiomics and ensemble methods.

The performance of predictions was assessed at four predetermined intervals: 6, 9, 12, and 15 months using a test dataset. In order to deepen the comparison between models, bootstrap random sampling was undertaken 100 times to facilitate statistical analysis. Table 4 outlines the C-index and AUC values across five distinct radiomics methods. Remarkably, the proportion delta radiomics method, adjusted for time, emerged as the superior performer, registering a C-index of 0.58 and a 12-month AUC of 0.65. These scores were notably higher than those achieved by other radiomics techniques. Meanwhile, Table 4 displays the C-index and AUC values for the clinical, radiomics, and ensemble methods when tested on the TVGH set. The evolution of time-dependent AUC values can be visualized in Figure 3. Notably, a combined strategy that harnessed both clinical data and delta-time radiomics attributes displayed a markedly enhanced performance relative to other methods. It achieved a C-index of 0.70. Furthermore, AUC values at intervals of 6, 9, 12, and 15 months were 0.74, 0.77, 0.78, and 0.78, respectively. Lastly, Table 4 provides the C-index and AUC values for the clinical, radiomics, and ensemble approaches on the combined test set. While the clinical features demonstrated consistent performance, there was a decrease in the C-index for the delta-time radiomics method by about 0.05. Similarly, the combined method also experienced a comparable decline.

**Figure 4:**
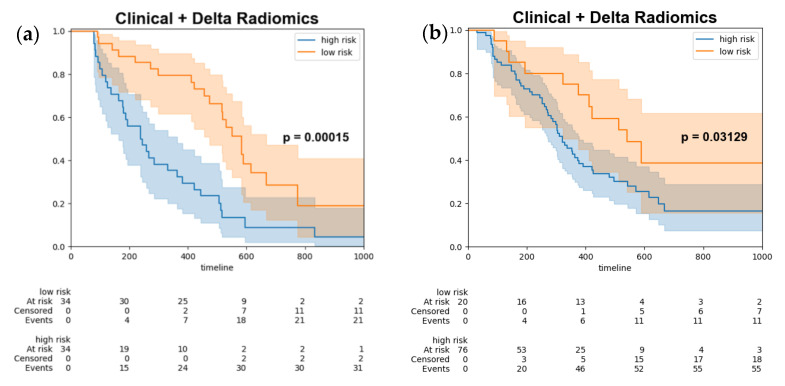
Kaplan–Meier survival curves of progression free survival stratify by model output (**a**) test set (**b**) combined test set. Kaplan-Meier survival curves for high-risk patients (blue line) and low risk patients (orange line). The shaded areas represent the 95% confidence intervals for each group.

Ultimately, the optimal threshold was determined by utilizing the Youden index derived from the training set, subsequently stratifying the test data (Figure 4a) and combined test set (Figure 4b) into high-risk and low-risk groups. The log-rank test was employed to assess the differences between the survival curves, yielding a *p*-value of 0.00015, which indicated a statistically significant disparity between the two survival groups.

## 4. Discussion

Our results demonstrated that delta time radiomics, when calculated using the percentage change method with time adjustment, showed superior performance compared to both simple percentage change of delta radiomics and log delta time radiomics approaches. This finding may be attributed to the fact that the percentage change method with time adjustment better captures the relative changes in tumor characteristics over time, accounting for both the baseline values, their changes during treatment, as well as the duration. The simple percentage delta radiomics method calculates the percentage difference between pre-treatment and follow-up radiomic features, whereas log-delta time radiomics uses a logarithmic transformation of the absolute differences. Although these methods provide some insight into the changes in tumor characteristics, they may not fully reflect the relative changes within the tumor, which could be of greater significance in predicting treatment response and patient prognosis. By contrast, the percentage change method with time adjustment considers the initial values of radiomic features and calculates the change as a proportion of the baseline values with time adjustment. This approach allows for the better normalization and scaling of features, making the results more comparable across different patients and tumors. In addition, the percentage change method with time adjustment may be more sensitive to subtle variations in tumor behavior, providing a more accurate representation of tumor dynamics and heterogeneity over time.

In the discussion of significant delta radiomic features, the selected features included LHL_Run_Length_Nonuniformity, LHH_Long_Run_Emphasis, and HLL_Variance. These features have been found to be important in capturing the underlying tumor characteristics, potentially aiding the prediction of treatment response and patient survival. LHL_Run_Length_Nonuniformity, for instance, is associated with the nonuniformity of run lengths in low gray-level regions, which may reflect the heterogeneity of tumor texture patterns [9]. This heterogeneity can be indicative of variations in cellularity, necrosis, and vascularization, all of which are crucial factors in determining tumor behavior and prognosis [8]. LHH_Long_Run_Emphasis, on the other hand, measures the distribution of long runs of high gray-level values, potentially indicating the presence of larger, more aggressive tumor regions [30]. This feature could be particularly relevant in understanding the spatial organization of tumor cells and the degree of invasiveness, ultimately impacting the choice of therapeutic strategies and patient management. Lastly, HLL_Variance reflects the variance in co-occurrence patterns of high and low gray-level values, providing insights into the spatial distribution of the tumor [31]. This feature may help reveal the underlying tumor microenvironment, including variations in stromal composition and immune cell infiltration, which are known to play a significant role in tumor progression and response to therapy [10]. Together, these significant delta radiomic features contribute to a comprehensive understanding of the tumor’s characteristics, enhancing the accuracy of prognostic models and informing more personalized therapeutic approaches.

The inclusion of clinical factors in our model, along with delta radiomics features, led to a significant improvement in prediction performance. This finding supports the notion that combining both clinical and imaging data can provide a more comprehensive and accurate representation of the tumor and its response to treatment [32]. In our study, we observed that the delta radiomics features did not exhibit a high correlation with the selected clinical factors. The low correlation between the delta radiomics and clinical features indicates that they provide complementary information about the tumor characteristics, which can enhance the predictive accuracy of the model when used together [14,33,34,35,36]. The delta radiomics features primarily capture changes in the tumor’s texture and spatial heterogeneity, while the clinical factors reflect the tumor’s biological and physiological properties [14].

The early detection of acquired resistance to EGFR-TKI therapy is fundamental in enhancing patient outcomes. This proactive detection, made possible through initial follow-up images after starting EGFR-TKI treatment, offers insights into the tumor’s response, be it regression, stabilization, or progression. These early radiographic signs enable clinicians to gauge the potential trajectory of a patient’s progression-free survival (PFS). Armed with this knowledge, they can then decide whether to maintain the current therapy, modify dosages, or switch to alternative treatments, ensuring the most effective approach for the patient. Furthermore, when delta radiomics is combined with clinical parameters such as AST, TP, PLT, N, and M staging, the resulting prognostic models are unparalleled in their predictive accuracy. This integrative methodology ensures that treatments are not only attuned to the patient’s cancer profile but also to their broader health context. In the realm of EGFR-TKI therapies, early awareness of acquired resistance is transformative. It goes beyond mere diagnosis, directly influencing the trajectory of patient care. Recognizing the initial signs, evident in follow-up images, equips clinicians with a unique perspective on the tumor’s behavior under treatment. This insight allows for data-driven, informed decisions, which not only elevate the quality of patient care but also prevent prolonged exposure to ineffective treatments, laying the groundwork for optimal clinical results.

In comparison to the study by Zhang X et al. [18], our research involved a larger cohort of 322 patients, which could contribute to more robust results. We also included contrast CT in our study, while the study by Zhang X et al. only included non-contrast CT. Additionally, we provided a detailed description of the selected radiomics features, furthering our understanding of their importance in predicting PFS in LUAD patients undergoing EGFR-TKI therapy. In our study, we also experimented with different formulas for calculating delta radiomics signatures. This allowed us to identify the optimal approach that yielded the best prognostic performance. In particular, we found that delta radiomics, especially the percentage change of radiomics with time adjustment, exhibited superior results compared to other methods. Moreover, our study demonstrated comparable prognostic performance to the aforementioned paper. Our results showed a c-index of 0.7 and a 12-month AUC of 0.78 in the testing set, which are in line with the reported c-index of 0.72 and 12-month AUC of 0.8 in the above paper. These findings further validate the potential of time-serial CT-based radiomics signatures as reliable biomarkers for predicting progression-free survival in lung adenocarcinoma patients undergoing EGFR-TKI therapy. By achieving comparable performance metrics, our study contributes to the growing body of evidence supporting the clinical applicability of delta radiomics in the management of these patients.

However, a moderate decline in delta radiomics and its combined approach with clinical methods was observed in the consolidated dataset. In contrast, the accuracy of clinical features remained consistent without any noticeable decline. This trend echoes findings from a previous study by Zhang X et al. [18]. Potential reasons for this decline in performance could include differences in CT hardware, such as manufacturers and models, between the initial and follow-up images, and even across different centers (Tables S2 and S3). Variations in imaging protocols, slice thickness, and in-plane resolution might also introduce heterogeneity. Although we incorporated isotropic resampling and normalization during preprocessing to mitigate these issues, further harmonization techniques could be essential. The differences due to hardware and protocols might have nonlinear relationships, and simplistic linear adjustments may not capture all heterogeneity nuances. Several studies have indicated that hardware and protocol variations can introduce inconsistencies in radiomic analyses [37,38,39,40,41,42,43]. Another potential factor could be that the inclusion of contrast-enhanced CT scans in the study might influence the extraction of radiomics, leading to potential inconsistencies in the data [37,38].

Despite its potential, the application of delta radiomics in clinical scenarios is not without challenges. One significant hurdle is the inconsistency in image acquisition protocols and scanner configurations. Such variability can manipulate the extracted radiomic features, thereby questioning the reproducibility and broad applicability of the results. It is evident that a standardized approach to imaging and a universal harmonization of radiomic features is the need of the hour for consistent clinical outcomes. Furthermore, the intricate nature of radiomics data often demands specialized computational methodologies and profound expertise, potentially restricting its broad-based clinical adoption, especially in settings with limited resources. The clinical pertinence of delta radiomics also mandates further validation through expansive, multi-institutional research to discern its tangible impact on patient prognoses [12].

Our research, while providing significant insights, is not devoid of its limitations. The retrospective design might impede the wider applicability of our conclusions. Addressing this limitation would require prospective studies spanning multiple centers, encompassing a larger and more heterogeneous patient demographic. This would permit a holistic evaluation of the clinical relevance and reliability of delta radiomics in evaluating NSCLC treatment responses. It is worth noting that in our research, a cohesive team of radiologists and certified pulmonologists undertook tumor segmentation on CT images. Incorporating automated segmentation methods could economize time and expenses linked with treatment strategizing and bolster the consistency in radiomic feature extraction.

## 5. Conclusions

In conclusion, our study demonstrates the potential of delta radiomics as a valuable tool for predicting treatment response and assessing the progression-free survival of patients with NSCLC undergoing EGFR-TKI therapy. The addition of the time variable to calculate delta radiomics provided more robust radiomics signatures. The incorporation of clinical factors alongside delta radiomics features improved the predictive performance of our model, indicating the importance of considering both imaging and clinical information for a comprehensive assessment. This comprehensive imaging signature has shown significant potential in predicting disease progression and enabling risk stratification. Once validated in larger cohorts, it can contribute to guiding clinical decision-making, such as the development of personalized follow-up strategies for patients with NSCLC. By harnessing the power of delta radiomics, clinicians can potentially enhance treatment planning and patient management, ultimately improving overall patient outcomes.

## Figures and Tables

**Table 1:** Characteristics of the 322 recruited NSCLC patients.

Characteristics	Training (*n* = 158)	Test (*n* = 68)	TCGH (*n* = 96)
Age			
<60, N (%)	70 (44.3)	22 (32.4)	29 (30.2)
>60, N (%)	88 (55.7)	46 (67.6)	67 (69.8)
Gender			
Female, N (%)	99 (62.7)	35 (51.5)	57 (59.4)
Smoking status			
Smoker, N (%)	35 (22.2)	23 (33.8)	21(21.9)
ECOG PS			
0, N (%)	49 (31.0)	35 (51.5)	4 (4.2)
1, N (%)	91 (57.6)	28 (41.2)	65 (67.7)
2, N (%)	11 (7.0)	4 (5.9)	10 (10.4)
>2, N (%)	7 (4.4)	1 (1.5)	17 (17.6)
Histology of NSCLC			
Adenocarcinoma, N (%)	155 (98.1)	65 (95.6)	89 (92.7)
Squamous cell carcinoma, N (%)	1 (1.3)	2 (2.9)	0 (0)
Other	2 (0.6)	1 (1.5)	7 (7.3)
Clinical T stage			
1, N (%)	19 (12.0)	10 (14.7)	10 (10.4)
2, N (%)	41 (25.9)	24 (35.3)	23 (24)
3, N (%)	27 (17.1)	9 (13.2)	6 (6.2)
4, N (%)	67 (42.4)	23 (33.8)	57 (59.4)
None	4 (2.5)	2 (2.9)	0 (0)
Clinical N stage			
0, N (%)	45 (28.5)	14 (20.6)	25 (26.0)
1, N (%)	13 (8.2)	6 (8.8)	2 (2.1)
2, N (%)	40 (25.3)	22 (32.4)	36 (37.5)
3, N (%)	59 (37.3)	25 (36.8)	33 (34.4)
None	1 (0.6)	1 (1.5)	0 (0)
Clinical M stage			
0, N (%)	6 (3.8)	2 (2.9)	8 (7.9)
1a, N (%)	47 (15.2)	24 (35.3)	35 (36.5)
1b, N (%)	24 (27.4)	8 (11.8)	53 (55.2)
1c, N (%)	81 (51.2)	34 (50.0)	
Clinical stage			
Stage IIIB, N (%)	14 (8.9)	5(7.4)	8 (8.3)
Stage IVA, N (%)	62 (39.2)	30 (44.1)	88 (91.7)
Stage IVB, N (%)	82 (51.9)	33 (48.5)	
EGFR mutation status			
Exon 19 deletion, N (%)	70 (44.3)	31 (45.6)	29 (30.2)
Exon 21 L858R substitution, N (%)	77 (48.7)	34 (50.0)	46 (47.9)
Others, N (%)	1 (7.0)	3 (4.4)	1(1)
None, N (%)	0 (0)	0 (0)	24 (25)
TKI			
Gefitinib, N (%)	29 (18.4)	9 (13.2)	NA
Erlotinib, N (%)	45 (28.5)	24 (35.3)	NA
Afatinib, N (%)	84 (53.2)	35 (51.5)	NA
Adverse drug reaction to EGFR-TKI			
Yes, N (%)	73 (46.2)	32 (47.1)	NA
Progress free survival, median(months)	12.4 (6.1–18.4)	13.9 (6.2–18.4)	10.5 (5.9–15.9)
Platelet			
median (IQR)	269,000 (230,250, 307,500)	269,000 (226,750–306,000)	277,000 (237,000–340,500)
Not available, N (%)	13 (8.2)	5 (7.4)	10 (10.4)
Aspartate aminotransferase			
median (IQR)	23 (18–27)	23 (18–27)	23 (19–28)
Not available, N (%)	60 (38)	24 (35.3)	19 (10.8)
Total protein			
median (IQR)	7.125 (6.8, 7.4)	7.075 (6.8, 7.4)	7.1 (6.8, 7.4)
Not available, N (%)	112 (70.9)	56 (82.4)	71 (74.0)

**Table 2:** Comparison of radiomics methods based on validation set.

PFS	Pretreatment Radiomics		Follow Up Radiomics		Delta Radiomics		Delta Time Radiomics		Delta log Time Radiomics	
	Train	Valid	Train	Valid	Train	Valid	Train	Valid	Train	Valid
C-index (95%CI)	0.55 (0.55–0.56)	0.55 (0.53–0.56)	0.58 (0.58–0.59)	0.56 (0.55–0.58)	0.63 (0.62–0.63)	0.57 (0.55–0.58)	0.63 (0.63–0.64)	0.58 (0.56–0.59)	0.58 (0.57–0.5)	0.57 (0.56–0.59)
t-AUC (95%CI)	0.56 (0.55–0.56)	0.55 (0.52–0.57)	0.59 (0.58–0.59)	0.56 (0.54–0.59)	0.65 (0.64, 0.65)	0.57 (0.54–0.59)	0.66 (0.66–0.67)	0.60 (0.58–0.62)	0.57 (0.56–0.57)	0.56 (0.54–0.58)

**Table 3:** Univariate and multivariate analysis of the final selected features.

Variable	Univariate		Multivariate	
	*p*-Value	HR (95%CI)	*p*-Value	HR (95%CI)
N1 vs. N0	0.02	2.43 (1.17–5.07)	<0.005	2.71 (1.48–4.95)
N2 vs. N0	0.24	1.38 (0.8–2.37)	0.12	1.42 (0.05–0.58)
N3 vs. N0	<0.005	2.24 (1.40–3.59)	<0.005	1.92 (1.28–2.87)
N None vs. N0	<0.54	1.57 (0.37–6.67)	0.28	2.21 (0.53–9.31)
M	0.01	1.29 (1.06–1.57)	<0.005	1.32 (1.12–1.56)
Platelet	<0.005	1.37 (1.11–1.70)	<0.005	1.36 (1.13–1.64)
Aspartate aminotransferase	<0.005	1.22 (1.07–1.38)	<0.005	1.31 (1.16–1.49)
Total protein	0.08	0.84 (0.7–1.02)	<0.005	1.32 (1.12–1.56)
LHL_Run_Length_Nonuniformity	0.03	4.46 (1.52–4960)	0.02	198 (1.73–22,709)
LHH_Long_Run_Emphasis	<0.005	14.39 (6817–4.63 × 10^8^)	<0.005	1.4 × 10^7^ (3.8 × 10^4^–5.1 × 10^9^)
HLL_Variance	<0.005	12.57 (2.19–72.13)	<0.005	14.19 (3.48–57.89)

**Table 4:** Statistical comparisons of different method on test dataset.

**(a): Statistical comparisons between developed prediction models with radiomics features based on test dataset**						
Test set (*n* = 100)	Model performance	Pre Rad (c-index = 0.53)	Follow Rad (c-index = 0.55)	Delta Rad (c-index = 0.56)	tDelta Rad (c-index = 0.58)	tDelta log Rad (c-index = 0.54)
6 month	Original AUC	0.57	0.53	0.53	0.61	0.51
	AUC	0.52 (0.51, 0.54)	0.54 (0.52, 0.56)	0.56 (0.54, 0.57)	0.62 (0.60, 0.62)	0.56 (0.54, 0.57)
9 month	Original AUC	0.46	0.53	0.57	0.63	0.51
	AUC	0.5 (0.49, 0.52)	0.53 (0.51, 0.55)	0.55 (0.53, 0.56)	0.62 (0.62, 0.65)	0.58 (0.57, 0.59)
12 month	Original AUC	0.53	0.50	0.56	0.65	0.53
	AUC	0.53 (0.51, 0.54)	0.50 (0.48, 0.51)	0.59 (0.58, 0.61)	0.65 (0.64, 0.67)	0.54 (0.53, 0.55)
15 month	Original AUC	0.58	0.58	0.55	0.67	0.55
	AUC	0.59 (0.58, 0.61)	0.58 (0.56, 0.59)	0.58 (0.56, 0.59)	0.67 (0.65, 0.68)	0.55 (0.54, 0.57)
	*p*-values					
	Pre rad vs. tDelta rad	Follow rad vs. tDelta rad	Delta rad vs. tDelta rad	tdelta log rad vs. tDelta rad		
6 month	<0.001 *	<0.001 *	<0.001 *	<0.001 *		
9 month	<0.001 *	<0.001 *	<0.001 *	<0.001 *		
12 month	<0.001 *	<0.001 *	<0.001 *	<0.001 *		
15 month	<0.001 *	<0.001 *	<0.001 *	<0.001 *		
**(b): Statistical comparisons between developed prediction models with the addition of clinical features based on test dataset**						
Test set (*n* = 100)	Model performance	Clinical (c-index = 0.66)	tDelta Rad (c-index = 0.58)	Clinical + tDelta Rad (c-index = 0.70)	*p*-values	
					Clinical vs Clinical + delta rad	delta rad vs clinical + delta rad
6 month	Original AUC	0.67	0.61	0.74	<0.001 *	<0.001 *
	AUC	0.68 (0.67~0.70)	0.62 (0.60, 0.62)	0.74 (0.73, 0.76)		
9 month	Original AUC	0.73	0.63	0.77	<0.001 *	<0.001 *
	AUC	0.74 (0.73~0.75)	0.62 (0.62, 0.65)	0.78 (0.77, 0.79)		
12 month	Original AUC	0.71	0.65	0.77	<0.001 *	<0.001 *
	AUC	0.72 (0.71~0.73)	0.65 (0.64, 0.67)	0.78 (0.77, 0.79)		
15 month	Original AUC	0.75	0.67	0.78	<0.001 *	<0.001 *
	AUC	0.76 (0.75~0.77)	0.67 (0.65, 0.68)	0.79 (0.78, 0.81)		
**(c): Statistical comparisons between developed prediction models with the addition of clinical features based on combined test dataset**						
Test set (*n* = 100)	Model performance	Clinical (c-index = 0.66)	tDelta Rad (c-index = 0.53)	Clinical + tDelta Rad (c-index = 0.64)	*p*-values	
					Clinical vs Clinical + delta rad	delta rad vs clinical + delta rad
6 month	Original AUC	0.73	0.53	0.68	<0.001 *	<0.001 *
	AUC	0.72 (0.71~0.73)	0.53 (0.52, 055)	0.68 (0.67, 0.70)		
9 month	Original AUC	0.72	0.59	0.73	0.003 *	<0.001 *
	AUC	0.67 (0.65~0.68)	0.59 (0.58, 0.60)	0.67 (0.66, 0.69)		
12 month	Original AUC	0.73	0.54	0.72	<0.001 *	<0.001 *
	AUC	0.71 (0.70~0.72)	0.53 (0.52, 0.54)	0.66 (0.65, 0.68)		
15 month	Original AUC	0.76	0.53	0.71	<0.001 *	<0.001 *
	AUC	0.73 (0.72~0.74)	0.54 (0.53, 0.55)	0.68 (0.66, 0.69)		

*: Statistically significant based on pair *t* test.

## Data Availability

Data available on request due to privacy and ethical restrictions.

## Supplementary Materials

The following supporting information can be downloaded at: https://www.mdpi.com/article/10.3390/cancers15215125/s1, Table S1: Formulae for the calculation of primary radiomic features; Table S2: CT Manufacturer and Model of TVGH dataset; Table S3: CT Manufacturer and Model of TCGH dataset. References [44,45,46,47,48,49] are cited in the supplementary materials.
