# Supplementary material for: Evaluating the Potential of Delta Radiomics for Assessing Tyrosine Kinase Inhibitor Treatment Response in Non-Small Cell Lung Cancer Patients
Source: Cancers (Basel). 2023 Oct 24;15(21):5125. doi: 10.3390/cancers15215125 (PMC10647242; doi:10.3390/cancers15215125)
Supplement: Supplementary file 1 [file cancers-15-05125-s001.zip › cancers-2647286-supplementary.pdf]

Supplementary Materials: Evaluating the Potential of Delta Radiomics for Assessing Tyrosine Kinase Inhibitor Treatment Response in Non-Small Cell Lung Cancer Patients

Ting-Wei Wang, Heng-Sheng Chao, Hwa-Yen Chiu, Yi-hui Lin, Hung-Chun Chen, Chia-Feng Lu, Chien-Yi Liao, Yen Lee, Tsu-Hui Shiao, Yuh-Min Chen, Jing-Wen Huang and Yu-Te Wu

Table S1. Formulae for the calculation of primary radiomic features.

Feature	Formula	Feature	Formula
**Intensity-based features (first-order statistics)**			
**X** denotes the intensity vector with N voxels of the tumor ROIs; $\overline{X}$, the mean of **X**; **P**, the first-order histogram with N_l discrete intensity levels.			
**Feature**	**Formula**	**Feature**	**Formula**
1. Energy	$\sum_{i=1}^{N} \mathbf{X}(i)^2$	2. Entropy	$\sum_{i=1}^{N_l} \mathbf{P}(i) log_2 \mathbf{P}(i)$
3. Kurtosis	$\frac{\frac{1}{N}\sum_{i=1}^{N}(\mathbf{X}(i)-\bar{X})^4}{\left(\sqrt{\frac{1}{N}\sum_{i=1}^{N}(\mathbf{X}(i)-\bar{X})^2}\right)^2} - 3$	4. Maximum	$\max(\mathbf{X})$
5. Mean	$\frac{1}{N}\sum_{i=1}^{N} \mathbf{X}(i)$	6. Mean absolute deviation	$\frac{1}{N}\sum_{i=1}^{N} \text{abs}(\mathbf{X}(i) - \bar{X})$
7. Median	$\text{median}(\mathbf{X})$	8. First quartile	Value that splits off the lowest 25% of data from the highest 75%
9. Third quartile	Value that splits off the highest 25% of data from the lowest 75%	10. Minimum	$\min(\mathbf{X})$
11. Range	$\max(\mathbf{X}) - \min(\mathbf{X})$	12. Root mean square (RMS)	$\sqrt{\frac{\sum_{i=1}^{N}\mathbf{X}(i)^2}{N}}$
13. Skewness	$\frac{\frac{1}{N}\sum_{i=1}^{N}(\mathbf{X}(i)-\bar{X})^3}{\left(\sqrt{\frac{1}{N}\sum_{i=1}^{N}(\mathbf{X}(i)-\bar{X})^2}\right)^3}$	14. Standard deviation	$\sqrt{\frac{1}{N}\sum_{i=1}^{N}(\mathbf{X}(i) - \bar{X})^2}$
15. Uniformity	$\sum_{i=1}^{N_l} \mathbf{P}(i)^2$	16. Variance	$\frac{1}{N}\sum_{i=1}^{N}(\mathbf{X}(i) - \bar{X})^2$
**Shape- and Size-based features**			
V, tumor volume; A, surface area of the volume			
17. Compactness 1	$\frac{V}{\sqrt{\pi}A^{3/2}}$	18. Compactness 2	$36\pi\frac{V^2}{A^3}$
19. Maximum 3D diameter	The largest pairwise Euclidean distance between voxels on the surface of the tumor volume.	20. Spherical disproportion	$\frac{A}{4\pi R^2}$
21. Sphericity	$\frac{\pi^{\frac{1}{3}}(6V)^{\frac{2}{3}}}{A}$	22. Surface area	$A = \sum_{i=1}^{Ns}\frac{1}{2}\lvert a_i b_i \times a_i c_i \rvert$<br>$Ns$, total number of triangles covering the surface; a, b, and c, triangle vertices
23. Surface to volume ratio	$\frac{A}{V}$	24. Volume	Number of pixels in the tumor region multiplied by the voxel size
**Textural features (gray-level co-occurrence matrix–based features)**			
$\mathbf{P}(\delta, \alpha)$, co-occurrence matrix for an arbitrary distance δ and direction α; N_g , number of discrete intensity levels in the image; p_x(i), marginal row probabilities; p_y(i), marginal column probabilities; μ_x, mean of p_x; μ_y, mean of p_y; σ_x, standard deviation of p_x; σ_y, standard deviation of p_y; HXY, entropy of **P**; HX, entropy of p_x; and HY , entropy of p_y;<br>$p_{x+y}(k) = \sum_{i=1}^{N_g}\sum_{j=1}^{N_g}\mathbf{P}(i,j), i + j = k, k = 2,3,\ldots,2N_g$;<br>$p_{x-y}(k) = \sum_{i=1}^{N_g}\sum_{j=1}^{N_g}\mathbf{P}(i,j), \lvert i - j\rvert = k, k = 0,1,\ldots,N_g - 1$;<br>$\text{HX} = -\sum_{i=1}^{N_g} p_x(i)\log_2(p_x(i)), \text{HY} = -\sum_{i=1}^{N_g} p_y(i)\log_2\left(p_y(i)\right)$;<br>$\text{HXY1} = -\sum_{i=1}^{N_g}\sum_{j=1}^{N_g}\mathbf{P}(i,j)\log_2(p_x(i)p_y(j))$, $\text{HXY2} = -\sum_{i=1}^{N_g}\sum_{j=1}^{N_g} p_x(i)p_y(j)\log_2(p_x(i)p_y(j))$			
25. Autocorrelation	$\sum_{i=1}^{N_g}\sum_{j=1}^{N_g} ij\mathbf{P}(i,j)$	26. Cluster Prominence	$\sum_{i=1}^{N_g}\sum_{j=1}^{N_g}\left[\boldsymbol{i} + \boldsymbol{j} - \boldsymbol{\mu_x} - \boldsymbol{\mu_y}\right]^{\mathbf{4}}\mathbf{P}(i,j)$
27. Cluster Shade	$\sum_{i=1}^{N_g}\sum_{j=1}^{N_g}\left[\boldsymbol{i} + \boldsymbol{j} - \boldsymbol{\mu_x} - \boldsymbol{\mu_y}\right]^{\mathbf{3}}\mathbf{P}(i,j)$	28. Cluster Tendency	$\sum_{i=1}^{N_g}\sum_{j=1}^{N_g}\left[\boldsymbol{i} + \boldsymbol{j} - \boldsymbol{\mu_x} - \boldsymbol{\mu_y}\right]^{\mathbf{2}}\mathbf{P}(i,j)$
29. Contrast	$\sum_{i=1}^{N_g}\sum_{j=1}^{N_g}\lvert\boldsymbol{i} - \boldsymbol{j}\rvert^{\mathbf{2}}\mathbf{P}(i,j)$	30. Correlation	$\sum_{i=1}^{N_g}\sum_{j=1}^{N_g}\frac{ij\mathbf{P}(i,j)-\mu_x(i)\mu_y(j)}{\sigma_x(i)\sigma_y(j)}$
31. Difference entropy	$\sum_{i=0}^{N_g-1} p_{x-y}(i) log_2\left[p_{x-y}(i)\right]$	32. Dissimilarity	$\sum_{i=1}^{N_g}\sum_{j=1}^{N_g}\lvert\boldsymbol{i} - \boldsymbol{j}\rvert\mathbf{P}(i,j)$
33. Energy	$\sum_{i=1}^{N_g}\sum_{j=1}^{N_g}[\mathbf{P}(i,j)]^{\mathbf{2}}$	34. Entropy (HXY)	$-\sum_{i=1}^{N_g}\sum_{j=1}^{N_g}\mathbf{P}(i,j)\log_2(\mathbf{P}(i,j))$
35. Homogeneity 1	$\sum_{i=1}^{N_g}\sum_{j=1}^{N_g}\frac{\mathbf{P}(i,j)}{1+\lvert i-j\rvert}$	36. Homogeneity 2	$\sum_{i=1}^{N_g}\sum_{j=1}^{N_g}\frac{\mathbf{P}(i,j)}{1+\lvert i-j\rvert^2}$
37. Informational measure of correlation 1	$\frac{HXY-HXY1}{\max(HX,HY)}$	38. Informational measure of correlation 2	$\sqrt{1 - e^{-2(HXY2-HXY)}}$

39. Inverse Difference Moment Normalized	$\sum_{i=1}^{N_g}\sum_{j=1}^{N_g}\frac{\mathbf{P}(i,j)}{1+\left(\frac{\lvert i-j\rvert^2}{N^2}\right)}$	40. Inverse Difference Normalized	$\sum_{i=1}^{N_g}\sum_{j=1}^{N_g}\frac{\mathbf{P}(i,j)}{1+\left(\frac{\lvert i-j\rvert}{N}\right)}$
41. Inverse variance	$\sum_{i=1}^{N_g}\sum_{j=1}^{N_g}\frac{\mathbf{P}(i,j)}{\lvert i-j\rvert^2}, i \neq j$	42. Maximum Probability	$\max(\mathbf{P}(i,j))$
43. Sum average	$\sum_{i=2}^{2N_g}\left[i\mathbf{P}_{x+y}(i)\right]$	44. Sum entropy	$-\sum_{i=2}^{2N_g}\mathbf{P}_{x+y}(i)log_2\left[\mathbf{P}_{x+y}(i)\right]$
45. Variance	$\sum_{i=1}^{N_g}\sum_{j=1}^{N_g}(i-\mu)^2\mathbf{P}(\mathbf{i},\mathbf{j})$		

Textural features (gray-level run-length matrix–based features)

$p(i,j|\theta)$, (*i,j*)th entry in the given run-length matrix p for a direction θ; N_g, number of discrete intensity levels in the image; and N_r, number of different run lengths

46. Short Run Emphasis	$\frac{\sum_{i=1}^{N_g}\sum_{j=1}^{N_r}\left[\frac{p(i,j\vert\theta)}{j^2}\right]}{\sum_{i=1}^{N_g}\sum_{j=1}^{N_r}p(i,j\vert\theta)}$	47. Long Run Emphasis	$\frac{\sum_{i=1}^{N_g}\sum_{j=1}^{N_r}j^2p(i,j\vert\theta)}{\sum_{i=1}^{N_g}\sum_{j=1}^{N_r}p(i,j\vert\theta)}$
48. Gray Level Non-Uniformity	$\frac{\sum_{i=1}^{N_g}\left[\sum_{j=1}^{N_r}p(i,j\vert\theta)\right]^2}{\sum_{i=1}^{N_g}\sum_{j=1}^{N_r}p(i,j\vert\theta)}$	49. Run Length Non-Uniformity	$\frac{\sum_{j=1}^{N_r}\left[\sum_{i=1}^{N_g}p(i,j\vert\theta)\right]^2}{\sum_{i=1}^{N_g}\sum_{j=1}^{N_r}p(i,j\vert\theta)}$
50. Run Percentage	$\sum_{i=1}^{N_g}\sum_{j=1}^{N_r}\frac{p(i,j\vert\theta)}{N_p}$	51. Low Gray Level Run Emphasis	$\frac{\sum_{i=1}^{N_g}\sum_{j=1}^{N_r}\left[\frac{p(i,j\vert\theta)}{i^2}\right]}{\sum_{i=1}^{N_g}\sum_{j=1}^{N_r}p(i,j\vert\theta)}$
52. High Gray Level Run Emphasis	$\frac{\sum_{i=1}^{N_g}\sum_{j=1}^{N_r}i^2p(i,j\vert\theta)}{\sum_{i=1}^{N_g}\sum_{j=1}^{N_r}p(i,j\vert\theta)}$	53. Short Run Low Gray Level Emphasis	$\frac{\sum_{i=1}^{N_g}\sum_{j=1}^{N_r}\left[\frac{p(i,j\vert\theta)}{i^2j^2}\right]}{\sum_{i=1}^{N_g}\sum_{j=1}^{N_r}p(i,j\vert\theta)}$
54. Short Run High Gray Level Emphasis	$\frac{\sum_{i=1}^{N_g}\sum_{j=1}^{N_r}\left[\frac{p(i,j\vert\theta)i^2}{j^2}\right]}{\sum_{i=1}^{N_g}\sum_{j=1}^{N_r}p(i,j\vert\theta)}$	55. Long Run Low Gray Level Emphasis	$\frac{\sum_{i=1}^{N_g}\sum_{j=1}^{N_r}\left[\frac{p(i,j\vert\theta)j^2}{i^2}\right]}{\sum_{i=1}^{N_g}\sum_{j=1}^{N_r}p(i,j\vert\theta)}$
56. Long Run High Gray Level Emphasis	$\frac{\sum_{i=1}^{N_g}\sum_{j=1}^{N_r}i^2j^2p(i,j\vert\theta)}{\sum_{i=1}^{N_g}\sum_{j=1}^{N_r}p(i,j\vert\theta)}$		

Textural features (local binary pattern–based features)

X denotes the vector of the local binary pattern with *N* voxels in tumor ROIs. The local binary pattern was estimated on the basis of the relations of the center pixel with eight neighbors; $\overline{X}$ is the mean of **X**, and **P** is the first-order histogram with N_l discrete intensity levels. Equations (1)–(16) (first-order statistics) were then applied to yield 16 local binary pattern–based features.

Table S2. CT Manufacturer and Model of TVGH dataset.

	CT Manufacturer Model	n
	GE MEDICAL SYSTEMS BrightSpeed	2
	GE MEDICAL SYSTEMS Brivo CT385 Series	1
	GE MEDICAL SYSTEMS Discovery CT	1
	GE MEDICAL SYSTEMS Discovery STE	1
	GE MEDICAL SYSTEMS LightSpeed VCT	12
	GE MEDICAL SYSTEMS Optima CT660	4
	GE MEDICAL SYSTEMS Revolution HD	2
	Hitachi Medical Corporation SCENARIA	1
	Philips Brilliance 64	32
	Philips iCT 256	31
	SIEMENS Emotion 16	1
	SIEMENS Emotion 16 (2010)	4
**Before**	SIEMENS Perspective	1
	SIEMENS SOMATOM Definition	1
	SIEMENS SOMATOM Definition AS	5
	SIEMENS SOMATOM Definition AS+	5
	SIEMENS SOMATOM Definition Flash	18
	SIEMENS Sensation 16	14
	SIEMENS Sensation 64	1
	SIEMENS syngo.via.VB20A	1
	TOSHIBA Aquilion	33
	TOSHIBA Aquilion ONE	1
	TOSHIBA Aquilion PRIME	14
	TOSHIBA Aquilion Prime SP	3
	Missing	61
	GE MEDICAL SYSTEMS LightSpeed VCT	17
	Philips Brilliance 64	4
	Philips iCT 256	68
**Follow**	SIEMENS Perspective	1
	SIEMENS SOMATOM Definition AS	9
	SIEMENS SOMATOM Definition Flash	29

SIEMENS Sensation 16	16
TOSHIBA Aquilion	39
TOSHIBA Aquilion PRIME	16
TOSHIBA Aquilion Prime SP	18
Missing	33

Table S3. CT Manufacturer and Model of TCGH dataset.

	CT Manufacturer Model	n
**Before**	GE MEDICAL SYSTEMS LightSpeed VCT	2
	GE MEDICAL SYSTEMS LightSpeed16	2
	GE MEDICAL SYSTEMS Optima CT660	8
	GE MEDICAL SYSTEMS Revolution CT	6
	INFINITT INFINITT PACS	1
	Philips Brilliance 64	36
	Philips iCT 256	29
	SIEMENS SOMATOM Definition AS	2
	SIEMENS SOMATOM Definition AS+	2
	TOSHIBA Aquilion	3
	TOSHIBA Aquilion ONE	1
	TOSHIBA Aquilion PRIME	3
	TOSHIBA Asteion	1
**Follow**	GE MEDICAL SYSTEMS Revolution CT	14
	Philips Brilliance 64	22
	Philips iCT 256	60

Exclusion criteria:

In studies focusing on NSCLC (non-small cell lung cancer) treatment, patient selection is vital. This is primarily due to the highly heterogeneous nature of lung cancer, especially at the molecular level. The exclusion criteria detailed below ensure the results obtained are specific to the target patient group, eliminating potential confounders.

1. **Molecular Mutations:**

Patients were excluded if they had mutations other than: EGFR (Epidermal Growth Factor Receptor) mutations, ALK (Anaplastic Lymphoma Kinase) fusions, KRAS (Kirsten Rat Sarcoma Viral Oncogene Homolog) mutations, BRAF (B-Raf Proto-Oncogene, Serine/Threonine Kinase) mutations

Explanation: Different molecular mutations have different roles in the pathogenesis and progression of NSCLC. These mutations also affect how a patient responds to treatment. For instance:

EGFR mutations are common in NSCLC and have been linked to increased sensitivity to EGFR-TKIs. They are found in 10-15% of Caucasian and up to 50% of Asian patients with NSCLC.

ALK fusions represent another targetable mutation, with specialized ALK inhibitors available for treatment. They occur in about 3-7% of NSCLC patients.

KRAS mutations are seen in 25-30% of NSCLC and historically have been challenging to target directly.

BRAF mutations occur in about 2-4% of NSCLC and can be targeted with BRAF inhibitors.

Patients with these other mutations may not benefit from the specific treatments under study or may confound the results due to variable responses.

2. **EGFR-TKI Treatment:**

Patients who received third-generation EGFR-TKIs as first-line therapy were excluded.

Explanation of EGFR-TKI Technology: EGFR-TKIs (Epidermal Growth Factor Receptor Tyrosine Kinase Inhibitors) are a class of targeted therapies designed to inhibit the EGFR tyrosine kinase, a protein that promotes cancer cell growth.

First-generation EGFR-TKIs (e.g., erlotinib, gefitinib): These are non-selective and bind reversibly to the ATP pocket of the tyrosine kinase domain. They have been shown to improve response rates and progression-free survival in patients with EGFR-mutant NSCLC.

Second-generation EGFR-TKIs (e.g., afatinib, dacomitinib): They bind irreversibly to the ATP pocket and have activity against first-generation TKI-resistant mutations. However, they still have limited activity against the T790M resistance mutation.

Third-generation EGFR-TKIs (e.g., osimertinib): They are designed to overcome resistance due to the T790M mutation, which emerges in many patients treated with first or second-generation TKIs.

Excluding patients who already received third-generation TKIs ensures the study can evaluate the efficacy of these drugs as a later line of therapy.

3. **Imaging and Follow-Up Data:**

Patients with no visible tumor lesions on images, insufficient follow-up information, or who had a follow-up CT scan not between 6-16 weeks were excluded. This ensures that there's measurable disease to evaluate the response to treatment and that follow-up data is available and consistent for analysis.

4. **Additional Exclusions:**

Patients with missing dosing time or clinical data, those who were lost to follow-up, had no lesion, experienced early death, or had early progression disease were also excluded to ensure data accuracy and integrity.

In conclusion, these exclusion criteria ensure that the patient group under study is homogenous, which is crucial for producing reliable and interpretable results in clinical research.

Summary of feature selection algorithms:
KBest: Select features according to the k highest scores.
LASSO: Linear Model trained with L1 prior as regularizer
Ridge: Linear least squares with l2 regularization.
Elastic net: Linear regression with combined L1 and L2 priors as regularizer.

Summary of machine learning algorithms:
CoxPH: The Cox proportional hazard model [1] incorporates elastic net regularization and is a semiparametric approach that models the hazard function by assuming a proportional relationship between its time component and feature component. Elastic net penalty combines the LASSO's subset selection property with the Ridge penalty's regularization strength [2].
Survival tree: A survival tree [3] is a tree-based technique for handling censored survival data, focusing on maximizing the survival difference between patient groups represented by nodes in a binary tree.
Random survival forest: The random survival forest [4] is an adaptation of the random forest method tailored for right-censored survival data analysis.
gradient-boosting machine A gradient-boosting machine [5] is a nonparametric model that employs an ensemble of regression trees to determine the hazard function's variation concerning associated covariates. The ensemble model is trained using a gradient-boosting technique to optimize the concordance index's smoothed approximation.
Fast support vector machine: The fast support vector machine [6] optimizes in the primal domain using truncated Newton optimization and leverages order statistic trees to reduce training's computational cost.

References

1. Friedman, J.; Hastie, T; Tibshirani, R Regularization paths for generalized linear models via coordinate descent. *J Stat Softw* **2010**, 33, 1–22.
2. Leblanc, M.; Crowley, J. Survival Trees by Goodness of Split. *J. Am. Stat. Assoc.* **1993**, 88, 457–467.
3. Ishwaran, H.; Kogalur, U.B.; Blackstone, E.H.; Lauer, M.S. Random survival forests. *Ann. Appl. Stat.* **2008** , 2, 841–860.
4. Chen, Y.; Jia, Z.; Mercola, D.; Xie, X. A gradient boosting algorithm for survival analysis via direct optimization of concordance index. *Comput Math Methods Med.* **2013**, 2013, 873595 https://doi.org/10.1155/2013/873595
5. Pölsterl, S.; Navab, N.; Katouzian, A. Fast Training of Support Vector Machines for Survival Analysis. *Mach. Learn. Knowl. Discov. Databases* **2015** , 9285, 243-259.
6. Håvard, K.; Borgan, Ø.; Scheel, I. Time-to-event prediction with neural networks and Cox regression. *J. Mach. Learn. Res.* **2019**, 20, 1–30.
